# Dr. Fairfield Snyder

**Published:** 1912-02

**Authors:** 


Dr. Fairfield Snyder, Buffalo 1872, died November 10, 1911 at Carunna, Ind., of cerebral hemorrhage, aged 64.

